# Correlations between TBL1XR1 and recurrence of colorectal cancer

**DOI:** 10.1038/srep44275

**Published:** 2017-03-15

**Authors:** Hongda Liu, Yunfei Xu, Qun Zhang, Kangshuai Li, Dawei Wang, Shuo Li, Shanglei Ning, Hui Yang, Weichen Shi, Zhaochen Liu, Yuxin Chen

**Affiliations:** 1 Department of General Surgery, Qilu Hospital Affiliated to Shandong University, Jinan, Shandong 250012, China; 2 Department of Respiratory Medicine, Jinling Hospital, School of Medicine, Nanjing University, Nanjing 210002, China; 3 Department of Physiology and Pathophysiology, Peking University Health Science Center, Beijing, 100191, China; 4 302 Military Hospital of China, Beijing 100000, China; 5 Department of Gastrointestinal Surgery, Qianfoshan Hospital Affiliated to Shandong University, Jinan, Shandong 250012, China; 6 Department of Breast Surgery, Qianfoshan Hospital Affiliated to Shandong University, Jinan, Shandong 250012, China

## Abstract

More than 25% localized CRC patients died from post-operative metastasis, and risk of metastasis varies among individuals due to the high heterogeneity of CRC. Therefore, figuring out potential biomarkers for disease recurrence would be invaluable to improve the follow-up efficiency and clinical treatment. Transducin (β)-like 1 X-linked receptor 1 (TBL1XR1) is a core component of the nuclear receptor corepressor complex, which functions as a repressive coregulatory factor for multiple transcription factors. The clinical significance of TBL1XR1 in CRC hasn’t been fully elucidated. In this study, we investigated the expression of TBL1XR1 in primary CRC tissues and liver metastases from TNM stage IV CRC patients, and found that its expression in primary tumor tissues was an independent prognostic factor for tumor recurrence. Thus, we enrolled another cohort including TNM stage I-III patients to further evaluate the relationship between TBL1XR1 expression and disease recurrence. Accordingly, high TBL1XR1 expression indicates poor disease-free survival of stage I-III CRC patients. Furthermore, we confirmed the importance of β-catenin signaling pathways in TBL1XR1-mediated CRC cell oncogenicity by clinical and cellular results. Our results emphasize the necessity of individual therapy decisions based on clinical biomarkers, especially for localized CRC patients who are not routinely treated with adjunctive chemotherapy.

Colorectal cancer is a significant health problem, which represents the third most common cancer worldwide^1^,^2^. CRC still shows significant morbidity and mortality despite significant improvement in surgical resection and advances in radiotherapy, immunotherapy and chemotherapy^3^. The clinical outcomes are diverse among patients even with similar clinicopathological parameters and treatments, because CRC is a biologically heterogeneous disease and includes dysfunctions of multiple proteins that control cell proliferation and survival^4^. Patients with the same clinical stage of CRC might have distinct molecular drivers and different prognosis. Therefore, a better understanding of the oncogenic activities and molecular markers underlying CRC is urgently needed, for both the prognosis prediction and novel therapeutic development.

TBL1XR1 is a core component of nuclear receptor corepressor (NCoR) and silencing mediator for retinoid and thyroid hormone receptors (SMRT) complex, which can mediate the transcription activity of various transcription factors. The expression^5^,^6^,^7^, subcellular localization^8^, and post-translational modifications^9^,^10^ of TBL1XR1 were all reported to be involved in malignancy development. As for the digestive cancers, TBL1XR1 overexpression was reported to be correlated with the poor prognosis of gastric cancer recently^11^,^12^. The clinical significance of TBL1XR1, especially its role in predicting disease recurrence in CRC hasn’t been systematically reported, although its cellular functions were partly revealed in SW480 cell line^13^.

In the current study, to elucidate whether and how TBL1XR1 is involved in the aggression and recurrence of CRC, we initially evaluated TBL1XR1 expression in primary tumor tissues and paired liver metastases from 47 stage IV patients with synchronous liver metastasis. The expression of TBL1XR1 in primary tumor tissues, but not in liver metastases, was correlated with the number of liver metastases. In addition, TBL1XR1 expression level in CRC tissues can act as an independent prognostic factor for cancer recurrence after primary surgery resection, whereas its expression in liver metastases showed no independent predictive significance.

Therefore, we further enrolled another cohort which included stage I-III patients to evaluate whether TBL1XR1 can be helpful in predicting metastasis and recurrence. Univariate and multivariate analyses showed that high expression of TBL1XR1 is an independent risk factor for post-operative recurrence of localized and regional CRC patients. Furthermore, we performed cellular studies combined with gene overexpression and knock-down methods, and biofunctional studies revealed that β-catenin was the key molecular in regulating TBL1XR1-mediated cell proliferation and invasion.

## Results

### Characteristics of patients in cohort I and their correlations with TBL1XR1 expression

**Figure 1:**
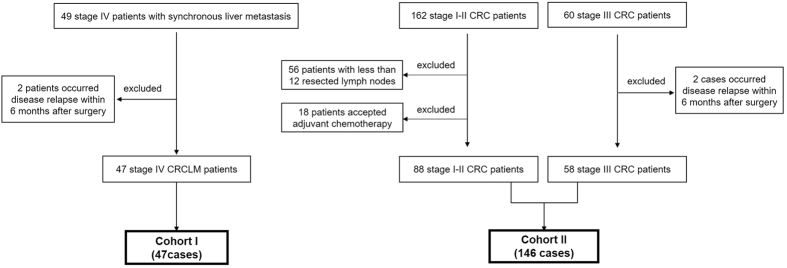
Patients selection. Cohort I included 47 TNM stage IV CRC patients with liver metastases (CRCLM). Cohort II included TNM stage I (19 cases), stage II (59 cases) and stage III (58 cases) patients, all of them underwent R0 resection.

The selection criteria for CRC patients were showed in Fig. 1. Briefly, we selected 47 stage IV CRC patients with synchronous liver metastasis (CRCLM) as cohort I to investigate the possible relationship between TBL1XR1 and CRC metastasis. Among them, 39 patients (83.0%) underwent R0 resection for both primary tumors and liver metastases, whereas the other 8 patients underwent R1 resection. Thirty-seven cases (78.7%) were treated with systematic adjunctive chemotherapy before detectable metachronous metastasis. Detailed clinicopathological characteristics of CRCLM patients were shown in Table 1.

**Figure 2:**
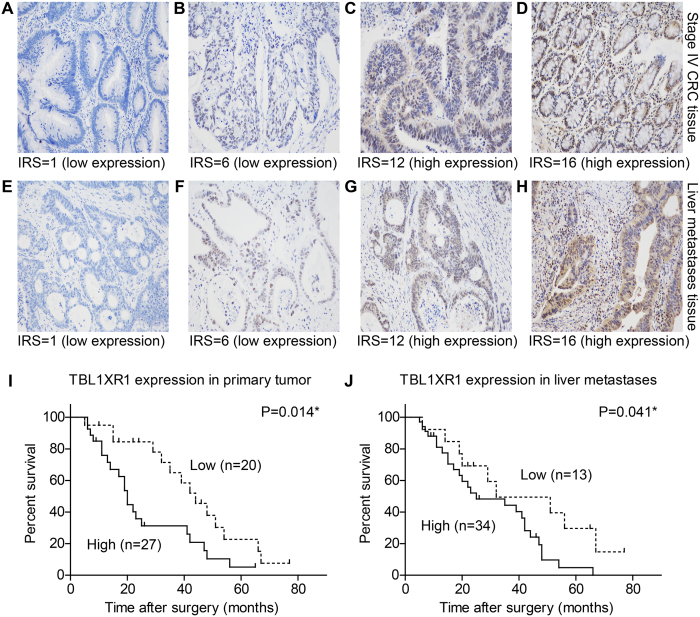
Expression of TBL1XR1 in stage IV CRCLM patients and its clinical significance. (**A**–**H**) Representative IHC results towards TBL1XR1 in TNM stage IV primary CRC tissues and liver metastases, showing different immunoreactivity score (IRS) among patients. (**A**,**B**) Low TBL1XR1 expression images in primary tumor tissues. (**C**,**D**) High TBL1XR1 expression in primary tumor tissues. (**E**,**F**) Low TBL1XR1 expression in liver metastases. (**G**,**H**) High TBL1XR1 expression in liver metastases. High expression of TBL1XR1 in primary tumor tissues (**I**) or liver metastases (**J**) were both correlated with poor disease-free survival (P = 0.014 and P = 0.041, respectively). Magnification: 200X. *Indicated statistical significance.

TBL1XR1 was identified as high expression in 27 primary CRC tissues (57.4%) and 34 liver metastases (72.3%), which was predominantly localized in the nucleus with slight immunoreactivity in cytoplasm of CRC cells (Fig. 2A–H). High expression of TBL1XR1 in primary tumor tissues was correlated with increasing number of liver metastases (P = 0.020, Table 1). However, we didn’t find any significant relationships between patients’ clinical features and TBL1XR1 levels in liver metastases. One possible explanation is that the protein patterns and functions in primary CRC cells may be more principle in determining the metastatic malignancy. Also, the microenvironment of tumor cells in liver metastases can be different compared with those in primary colorectal locations, which will significantly alter the expression and modifications of proteins.

### TBL1XR1 expression in primary tumor tissues is an independent predictive factor for DFS of patients in cohort I

We retrospectively achieved the information about tumor recurrence of the patients in cohort I. Totally 35 cases occurred metachronous metastasis, and the 3-year disease-free survival (DFS) was 45.8%. According to the Kaplan-Meier survival curves and log-rank test, we identified that high expression of TBL1XR1 in either primary tumor tissues (P = 0.014, Fig. 2I) or liver metastases (P = 0.041, Fig. 2J) was correlated with poor DFS. In addition, bilobar distribution of liver metastases (P = 0.043), R1 resection margin (P = 0.031) and absent of adjunctive chemotherapy (P = 0.007) were all unfavorable factors for DFS of CRCLM patients (Table 2).

Multivariate analysis with Cox regression hazard model was performed to better verify the significance of TBL1XR1 (Table 3), which demonstrated that its expression in primary CRC tissues was an independent prognostic factor for DFS of stage IV CRC patients (HR = 2.49, 95% CI 1.14–5.42, P = 0.022). Other significant hazard factors included liver metastases distribution (HR = 2.48, 95% CI 1.04–5.91, P = 0.041), surgery resection margin (HR = 4.09, 95% CI 1.34–12.47, P = 0.022) and post-operative chemotherapy (HR = 2.48, 95% CI 1.02–6.00, P = 0.044).

### Clinicopathological features of patients in cohort II

TBL1XR1 expression was shown to be correlated with the disease recurrence of the stage IV CRC patients according to the results from cohort I study. Therefore, we were interested to further investigate the possible role of TBL1XR1 in predicting metachronous metastasis for localized and regional CRC patients, which was critical in improving clinical decisions.

The cohort II patients included 19 cases with TNM stage I, 59 cases with stage II and 58 patients with stage III (Fig. 1). None of the stage I-II patients received any chemotherapy before detectable recurrence, whereas 46 stage III cases underwent routinely chemotherapy with fluorouracil and cisplatin after primary surgery resection. Totally, 8 cases occurred LN metastasis, 2 cases occurred lung metastasis, and 29 cases occurred liver metastasis during our follow-up. Other patients’ characteristics were shown in Table 4.

### Expression of TBL1XR1 in cohort II patients and its clinical significance

**Figure 3:**
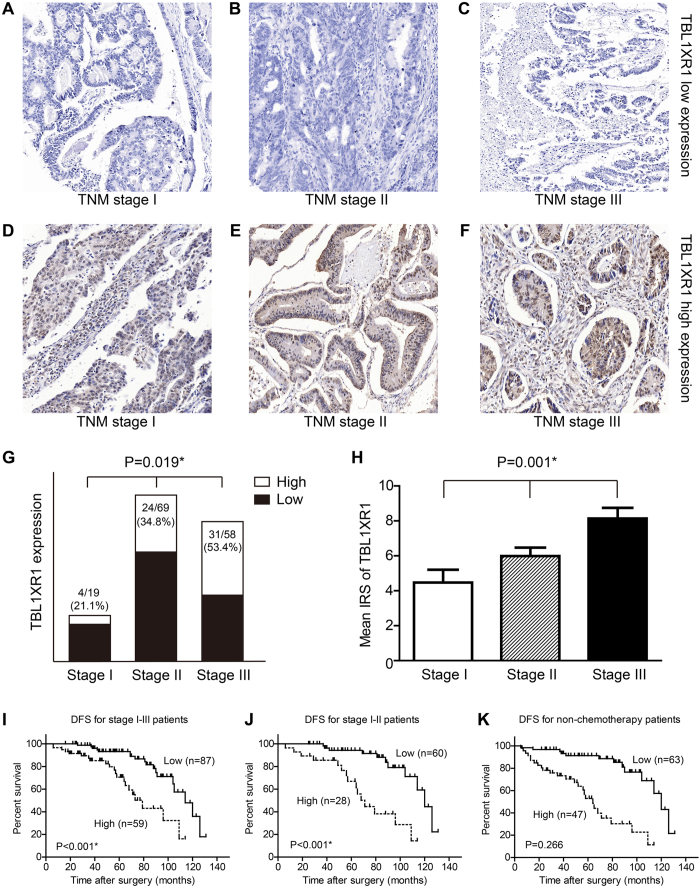
Expression of TBL1XR1 in stage I-III CRC patients and its clinical significance for disease-free survival. Reprehensive low expression (**A**–**C**) and high expression (**D**–**F**) of TBL1XR1 in tumor tissues from TNM stage I-III patients, respectively. (**G**) Patients with advanced TNM stage showed higher proportions of TBL1XR1 high expression (chi-square test, P = 0.019). (**H**) Quantification analyses of mean TBL1XR1 expression levels in tumor tissues from patients with different clinical stages. (**I**) Kaplan-Meier survival curves comparing cumulative disease-free survival rates in stage I-III patients with low and high TBL1XR1 expression levels. (**J**) Survival curves for CRC patients with clinical stage I-II. (**K**) Survival curves for non-chemotherapy CRC patients with TNM stage I-IV. Magnification: 200X. *Indicated statistical significance.

The expression pattern of TBL1XR1 in cohort II patients were revealed by IHC (Fig. 3A–F). The high expression ratio of TBL1XR1 was 21.1%, 34.8% and 53.4% for stage I-III CRC patients, respectively (Fig. 3G). Moreover, the mean immunoreactivity score (IRS) of TBL1XR1 was positively correlated with clinical stage (Fig. 3H), indicating that patients with more advanced TNM stage showed higher TBL1XR1 expression level.

We also performed the survival analysis for cohort II patients, which demonstrated that high expression of TBL1XR1 can indicate high recurrence risk of stage I-III CRC patients (Fig. 3I, Table 5) from both univariate and multivariate analyses (HR = 3.716, 95% CI 1.858–7.433, P < 0.001). Similarly, TBL1XR1 was significant in predicting metachronous metastasis for stage I-II subgroup patients (P < 0.001, Fig. 3J, Supplemental Table 1).

However, for the stage III subgroup, TBL1XR1 showed no statistical significance in DFS evaluation (P = 0.266, Supplemental Table 2). This perhaps due to the fact that most of the stage III patients underwent chemotherapy (46/58 cases, 79.3%), which was critical in inhibiting post-operative metastasis. To eliminate the effects of chemotherapy, we further evaluate the DFS patterns of the patients without adjunctive chemotherapy in both cohort I and cohort II. The Kaplan-Meier survival analysis showed that high expression of TBL1XR1 can significantly increase the recurrence risk of non-chemotherapy treated patients (P < 0.001, Fig. 3K, Supplemental Table 3).

### TBL1XR1 promotes cell proliferation and invasion by up-regulating the transcription activity of β-catenin

**Figure 4:**
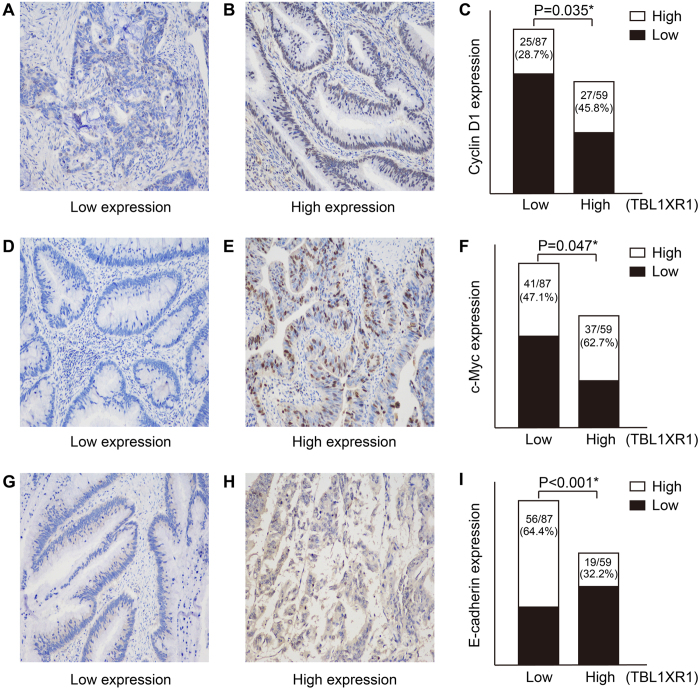
Correlations between TBL1XR1 expression and β-catenin down-stream signaling molecular levels in tumor tissues. Images showed the reprehensive low expression (**A**) and high expression (**B**) of Cyclin D1 in CRC tissues, and chi-square test demonstrated that the mean Cyclin D1 levels were higher in patients with high TBL1XR1 expression. (**D**–**F**) Similar results were also observed during the analyses for c-Myc protein levels by IHC and chi-square test. (**G**–**I**) In contrast, the protein levels of E-cadherin showed negative correlation with TBL1XR1 levels (P < 0.001), revealing the oncogenic role of TBL1XR1 in CRC development and aggression. Magnification: 200X. *Indicated statistical significance.

Interestingly, we found that TBL1XR1 expression was positively correlated with the Cyclin D1 (P = 0.035, Fig. 4A–C) and c-Myc levels (P = 0.047, Fig. 4D–F) in CRC tissues, both are well-known β-catenin-regulated proteins. In contrast, E-cadherin level was negatively correlated with TBL1XR1 level (P < 0.001, Fig. 4G–I). Thus, we further studied the cellular functions of TBL1XR1 in regulating CRC oncogenicity.

**Figure 5:**
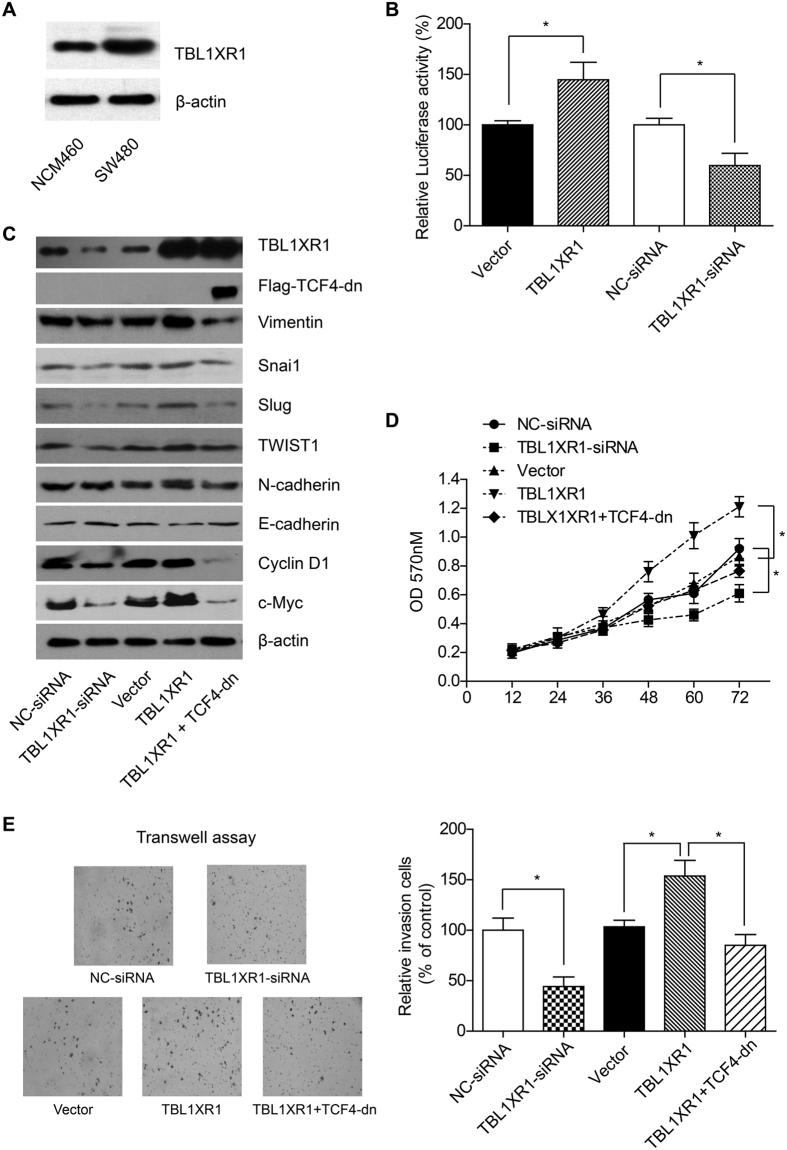
TBL1XR1 can promote EMT through β-catenin, therefore up-regulates cell proliferation and invasion. (**A**) The expression level of TBL1XR1 in SW480 cells was higher than that in NCM460 cells (normal epithelial cell line), which was consistent with the RT-qPCR and IHC results. (**B**) Overexpression of TBL1XR1 in SW480 cells can increase the transcription activity of β-catenin, while TBL1XR1 knock-down showed opposite effect. (**C**) TBL1XR1 overexpression can up-regulate the protein level of Vimentin, Snail, TWIST1, and N-cadherin, while the E-cadherin expression was down-regulated. On the other hand, competitive inhibition of β-catenin can impair the EMT protein expression. MTT assay (**D**) and Transwell assay (**E**) showed that TBL1XR1 can regulate the proliferation and invasion capacities of SW480 cells through Wnt-β-catenin signaling pathway.

TBL1XR1 showed higher expression level in SW480 cells than that in normal colon epithelial NCM480 cells (Fig. 5A). To elucidate the underlying mechanisms of TBL1XR1 in promoting CRC development, we tested the effect of TBL1XR1 overexpression and knock-down in SW480 CRC cells. Luciferase assay demonstrated that overexpression of TBL1XR1 can significantly enhance the transcription activity of β-catenin (Fig. 5B).

The mutation and continuous activation of β-catenin in CRC has been well-investigated^14^,^15^,^16^, therefore, the role of TBL1XR1 in regulating Wnt pathway may be mediated by cross-talk with β-catenin. Our results showed that TBL1XR1-siRNA can down-regulate the level of EMT proteins, such as Vimentin, Snai1, Slug, Twist1, and N-cadherin (Fig. 5C). Meanwhile, the expression of Cyclin-D1 and c-Myc were also inhibited under TBL1XR1-siRNA. On the other hand, overexpression of TBL1XR1 in SW480 cells can up-regulate the expression of Vimentin, Slug, c-Myc, etc.

We next transfected the inactive β-catenin mutant, TCF-4-dn, to better verify the participate of β-catenin in regulating TBL1XR1 signaling pathway. Western Blot showed that both β-catenin-target proteins and EMT proteins were less expressed in the cells co-transfected with TBL1XR1 and TCF-4-dn, compared with the cells only overexpressed TBL1XR1 (Fig. 5C). In addition, overexpression of TBL1XR1 can dramatically enhance cell proliferation and invasion capacities, while co-transfection with TCF4-dn can almost abolish the oncogenic effects of TBL1XR1 (Fig. 5D,E).

## Discussion

Most the CRC patients in the localized stages (TNM stage I and II) can be treated effectively by surgical resection with the 5-year OS up to 95% for stage I and 60–80% for stage II, respectively. However, the majority of patients with metastatic colorectal cancer in the advanced stages (TNM stage III and IV) have quite a poor prognosis since the 5-year OS drop dramatically to 35% with regional stage (stage III) and to 10% with distant stage (stage IV)^17^,^18^. Moreover, up to 25% of localized CRC patients ultimately die due to post-operative metastasis^19^. Thus, metastasis is the decisive and the most lethal event during this disease course. Molecular biomarker for cancer may be helpful for prediction of metastasis or therapeutic treatment^20^.

In this study, we explored the expression pattern of TBL1XR1 in CRC patients with different clinical stages (TNM I-IV), including both the primary tumor tissues and liver metastases. Consequently, we found that TBL1XR1 expression in primary CRC tissues can significantly help to predict disease relapse and recurrence after surgery for stage I-II and stage IV patients. However, the expression of TBL1XR1 showed no statistical significance for the prognosis of stage III CRC patients. This difference can be explained by discussing the distinct characteristics and clinical treatment for different stages.

Most of the stage I-II patients, which are lymph-node negative, don’t receive any post-operative adjunctive chemotherapy in clinical practice. Therefore, endogenous protein alterations are the predominant factors affecting the prognosis for those localized CRC patients. Liver is the most frequent metastasis localization for stage IV patients due to the near distance and sufficient circulation drainage. Although the predominant prognostic factor for stage IV CRC patients can be the status of metastasis, the biological changes in primary CRC determine the metastasis and recurrence pattern^21^. In contrast, stage III patients are more likely to suffer from metachronous metastasis or recurrence. However, the recurrence rate of stage III patients is not higher than that of stage II CRC patients during our follow-up due to the application of systematic post-operative chemotherapy. In fact, stage III patients were advised for routinely chemotherapy while stage I-II patients were not. Therefore, we suspect that current chemotherapy strategies may partly inhibit the TBL1XR1-regulated tumor aggression because we didn’t find clinical significance of TBL1XR1 in stage III patients. On the other hand, it was reported that TBL1XR1 can suppress cisplatin sensitivity in nasopharyngeal carcinoma^22^. Whether TBL1XR1 is involved in drug resistance for CRC patients and its potential in novel chemotherapy development remain to be further investigated. Anyway, we verified that TBL1XR1 was correlated with high recurrence risk for those patients who didn’t receive systematic adjunctive chemotherapy after surgery. Taken together, our clinical findings emphasized the clinical significance of TBL1XR1 expression in CRC patients with different clinical stages.

Although the involvement of TBL1XR1 in various malignancies has been identified, the functional study about how it participates in metastasis of CRC is poorly understand. Our cellular studies revealed that TBL1XR1 can up-regulate the transcription activity of β-catenin. Taking into consideration that SW480 cells possess APC mutation and high basal β-catenin level^23^, we transfected the inactive mutant of β-catenin (TCF-4-dn) to competitively inhibit its activity. Overexpression of TBL1XR1 can promote EMT and cell oncogenicity, while inhibition of β-catenin impaired its effect. Therefore, the tumor-promoting effect of TBL1XR1 is at least partially mediated through regulating the transcription activity of β-catenin.

## Conclusion

Our study not only identified TBL1XR1 as an independent prognostic factor for the recurrence of CRC patients, but also emphasized the potential of biomarker identification as the guidance for chemotherapy treatment of early stage CRC patients.

## Patients and Methods

### Patients selection

As shown in Fig. 1, we initially selected 49 CRC patients with synchronous liver metastasis who underwent surgical resection for both primary tumor tissues and liver metastases in Qilu Hospital, Qianfoshan Hospital, Shanxian Central Hospital, or Yuncheng People’s Hospital between 2000 to 2013. Among them, 2 patients occurred disease relapse with in the first 6 months after surgery and were excluded. Finally, 47 stage IV CRCLM patients were enrolled as cohort I in this study.

Another 162 stage I-II CRC patients were collected at the beginning. However, we later found that 56 patients among them were resected with less than 12 lymph nodes, which may increase the false negative possibility of lymph node metastasis. The World Congress of Gastroenterology proposed examination of a minimum of 12 lymph nodes for classification of tumors as Stage II^24^. Another 18 patients among them received post-operative chemotherapy. Therefore, both the 56 patients and 18 patients were excluded to better evaluate the clinical significance of TBL1XR1. In addition, 60 cases of stage III CRC patients were collected while later 2 patients were identified with disease relapse within 6 months after surgery, who were also excluded from our study. Finally, the cohort II group included 88 stage I-II and 58 stage III CRC patients (Fig. 1).

This study was approved by the Ethics Committee of Shandong University. Written informed consents were obtained from all the patients. The design of this study is accordance with the guidelines of CONSORT Statement (http://www.consort-statement.org/).

### IHC and IHC evaluation

Paraffin-embedded specimens were cut into 5-μm sections, followed by IHC staining with anti-TBL1XR1 (1:400, HPA019182, Sigma), anti-Cyclin-D1 (1:200, 60186-1-Ig, Proteintech), anti-c-Myc (1:200, 10828-1-AP, Proteintech) and anti-E-cadherin (1:400, 20874-1-AP, Proteintech) antibodies using the method described before^25^. IHC results were evaluated by two independent pathologists, according to both the staining intensity and percentage of positively stained cells. The staining intensity was defined as follows: 1 (negative staining), 2 (weak staining, slight yellow), 3 (moderate staining, dark yellow), 4 (strong staining, dark brown). The percentage of stained cells was scored as 1 (0–20% positive), 2 (21–50% positive), 3 (51–75% positive), 4 (76–100% positive). The final immunoreactivity score (IRS) was calculated by multiplying the average intensity score and percentage score (range 1–16), and protein expressions were identified as low-expression (IRS ≤ 6) and high-expression (IRS > 6) accordingly.

### Cell culture and transfection

Human CRC cell line SW480 was purchased from the American Type Culture Collection (ATCC, Manassas, VA, USA), and the NCM460 normal colonic epithelial cell line was obtained from Jennio Biotechnology (Guangzhou, China). SW480 cells were cultured in Dulbecco’s modified Eagle’s medium (DMEM) supplemented with 10% fetal bovine serum (FBS), 100 U/mL penicillin and 100 μg/mL streptomycin. NCM460 cells were maintained in RPMI 1640 medium with 10% FBS, 100 U/mL penicillin and 100 μg/mL streptomycin. All cells were cultured at 37 °C with 5% CO2.

pcDNA3.0-TBL1XR1 construct was generated by subcoloning the PCR-amplified human TBL1XR1 coding sequence into pcDNA3.0 vector. TCF4-dn in pLX303-vector was a gift from William Hahn (Addgene plasmid # 42592)^26^, and we constructed it with an additional Flag-tag. The siRNA sequence for TBL1XR1 was 5′-AAGGCCCUAUAUUUGCAUUAA-3′^27^, and the scramble siRNA sequence was 5′-UUCUCCGAACGUGUCACGU-3′. Both the transient overexpression and siRNA knock-down were carried out using Lipofectamine 2000 (Invitrogen) according to the manufacturer’s instructions.

### Western Blot

Western Blot was performed to evaluate the protein levels as describe before^28^. Briefly, cells were homogenized with NP-40 lysis buffer and then centrifuged the lysate at 12,000 rpm for 20 min to obtain supernatant. After protein concentration was determined by BCA protein assay kit (Thermo), equal amounts of total protein were resolved by 12% SDS-PAGE followed by transferring to PVDF membranes. Blots were blocked for 1 h in 5% BSA and then incubated overnight with the primary antibodies: TBL1XR1 (HPA019182, Sigma), Vimentin (10366-1-AP, Proteintech), Snai1 (13099-1-AP, Proteintech), Slug (12129-1-AP, Proteintech), TWIST1 (18125-1-AP, Proteintech), N-cadherin (13769-1-AP, Proteintech), E-cadherin (20874-1-AP, Proteintech), Cyclin D1 (60186-1-Ig, Proteintech), c-Myc (10828-1-AP, Proteintech) and β-actin (sc-1616, Santa Cruz). After washing three times with TBST, the membranes were incubated for another 1 h with secondary antibodies conjugated with horseradish peroxidase. The immunoreactivity was visualized using the enhanced chemiluminescence (ECL) reagent and autoradiographic film.

### Luciferase assay

pcDNA3.0-TBL1XR1 or TBL1XR1-siRNA transfected SW480 cells were seeded in 24-well plates. After 24 h culture, β-catenin responsive firefly Luciferase reporter plasmids and Renilla reporter plasmids (pRL-TK) were co-transfected into the cells. After another 16 h culture, the cells were stimulated with Wnt3a (200 ng/mL) for 6 h. Then lysed the cells and measured both Luciferase and Renilla activities with Dual-Luciferase Reporter Assay kit (Promega) according to the manufacturer’s instruction. The final Luciferase activity was normalized to the Renilla activity. Each experiment was performed in triplicate and repeated at least three times.

### Proliferation assay

Transfected cells were seeded into a 96-well plate, cultured for 24 h and then incubated with Wnt3a (200 ng/mL). At designated time points (12, 24, 36, 48, 60, and 72 h), 20 μL of 5 mg/mL 3-[4,5-dimethyl-2-thiazolyl]-2,5-diphenyl-2H-tetrazolium bromide (MTT) reagent was added into the wells and incubated for a further 4 h, then removed the medium, and added 150 μL DMSO subsequently to fully solubilize the MTT. The absorbance values were measured at 570 nm wavelength. Each experiment was performed in triplicate and repeated at least three times.

### Invasion assay

Transfected SW480 cells were subjected to a Transwell migration assay. 1 × 105 cells were planted onto the upper chamber and cultured with DMEM containing 10% FBS for 4 h, then replaced the medium with FBS-free DMEM. The lower chamber was filled with DMEM containing 10% FBS. After incubation for 24 h, cells in the upper surface of membrane were carefully removed with a cotton swab, and cells that invaded to lower surface were fixed and stained with 0.5% crystal violet. The number of invading cells was counted from five randomly selected visual fields.

### Statistics

Disease free survival (DFS) was defined as the period from tumor resection to the date of recurrence (including local recurrence, regional recurrence and distant recurrence) or till the date of our last follow-up. Data analyses were performed with SPSS 20.0 and Graphpad Prism 5.0 software. The correlations between TBL1XR1 expression with patients’ clinicopathological characteristics were evaluated with chi-square test. The quantitively IRS comparison among patients with different TNM stages were carried out by one-way ANOVA analysis. Kaplan-Meier survival analysis and log-rank test were used to identify the prognostic value of various parameters, and the factors with statistical significance were further subjected to multivariate analysis by Cox regression hazard model. A P < 0.05 was considered as statistical significant (two-tailed).

## Additional Information

**How to cite this article**: Liu, H. *et al*. Correlations between TBL1XR1 and recurrence of colorectal cancer. *Sci. Rep.* **7**, 44275; doi: 10.1038/srep44275 (2017).

**Publisher's note:** Springer Nature remains neutral with regard to jurisdictional claims in published maps and institutional affiliations.

## Supplementary Material

Supplemental Tables

## Figures and Tables

**Table 1:** Correlations between TBL1XR1 expression in tumor tissues and clinical characteristics of stage IV CRCLM patients (Cohort I).

Variables	Cases (n = 47)	TBL1XR1 expression in primary CRC tissues		χ^2^ test P value	TBL1XR1 expression in liver metastases		χ^2^ test P value
		Low (n = 20)	High (n = 27)		Low (n = 13)	High (n = 34)	
Gender				0.134			0.312
Female	20	6	14		4	16	
Male	27	14	13		9	18	
Age (year)				0.886			0.248
<60	17	7	10		3	14	
≥60	30	13	17		10	20	
Primary tumor location				0.095			0.245
Colon	34	17	17		11	23	
Rectum	13	3	10		2	11	
Primary tumor differentiation				0.381			0.422
Poor	15	5	10		3	12	
Well/Moderate	32	15	17		10	22	
CRCLM distribution				0.726			0.245
Unilobar	34	15	19		11	23	
Bilobar	13	5	8		2	11	
No. of CRCLM				0.020*			0.768
<3	34	18	16		9	25	
≥3	13	2	11		4	9	
Resection Margin				0.270			0.495
R0	39	18	21		10	29	
R1	8	2	6		3	5	
Adjunctive Chemotherapy				0.366			0.159
Yes	37	17	20		12	25	
No	10	3	7		1	9	

Abbreviations: TBL1XR1, Transducin (β)-like 1 X-linked receptor 1; CRC, colorectal cancer; CRCLM, colorectal cancer liver metastases.

**Table 2:** DFS of stage IV patients with CRCLM (Cohort I).

Variables	Cases (n = 47)	3-year DFS (%)	DFS (months) Mean ± S.D.	P value
Gender				0.515
Female	20	37.0%	31.2 ± 4.9	
Male	27	53.0%	35.5 ± 4.3	
Age (year)				0.672
<60	17	62.0%	37.1 ± 4.5	
≥60	30	38.5%	32.1 ± 4.3	
Primary tumor location				0.082
Colon	34	48.5%	36.6 ± 3.9	
Rectum	13	41.0%	26.2 ± 4.8	
Primary tumor differentiation				0.332
Poor	15	59.3%	38.5 ± 5.8	
Well/Moderate	32	38.3%	30.9 ± 3.8	
CRCLM distribution				0.043*
Unilobar	34	53.1%	37.6 ± 3.5	
Bilobar	13	25.4%	22.9 ± 6.3	
No. of CRCLM				0.253
<3	34	50.7%	36.2 ± 3.5	
≥3	13	32.1%	28.0 ± 7.9	
Resection Margin				0.031*
R0	39	50.4%	36.1 ± 3.4	
R1	8	0.0%	15.3 ± 2.6	
Adjunctive chemotherapy				0.007*
Yes	37	52.7%	37.4 ± 3.6	
No	10	17.1%	19.3 ± 4.4	
TBL1XR1 expression in CRC				0.014*
Low	20	65.0%	43.7 ± 4.7	
High	27	30.9%	25.9 ± 3.6	
TBL1XR1 expression in CRCLM				0.041*
Low	13	49.5%	42.5 ± 7.0	
High	34	44.0%	30.1 ± 3.3	

Abbreviations: DFS, disease-free survival; TBL1XR1, Transducin (β)-like 1 X-linked receptor 1; CRC, colorectal cancer; CRCLM, colorectal cancer liver metastases.

**Table 3:** Multivariate analysis of stage IV patients with CRCLM (Cohort I).

Variable	HR	95% CI	P value
CRCLM distribution			
Bilobar	2.48	1.04~5.91	0.041*
Unilobar	Reference		
Resection Margin			
R1	4.09	1.34~12.47	0.013*
R0	Reference		
Adjunctive chemotherapy			
No	2.48	1.02~6.00	0.044*
Yes	Reference		
TBL1XR1 expression in CRC			
High	2.49	1.14~5.42	0.022*
Low	Reference		
TBL1XR1 expression in CRCLM			
High	2.07	0.85~5.04	0.110
Low	Reference		

Abbreviations: HR, hazard ratio; CI, confidential interval; TBL1XR1, Transducin (β)-like 1 X-linked receptor 1; CRCLM, colorectal cancer liver metastases; CRC, colorectal cancer.

**Table 4:** Expression of TBL1XR1 in TNM stage I-III CRC patients (Cohort II).

Variables	Cases (n = 146)	TBL1XR1 expression		χ^2^ test P value
		Low (n = 87)	High (n = 59)	
Gender				0.177
Female	50	26	24	
Male	96	61	35	
Age (year)				0.399
<60	73	46	27	
≥60	73	41	32	
Preoperative CEA level				0.664
<100 ng/ml	70	43	27	
≥100 ng/ml	76	44	32	
Tumor location				0.565
Colon	98	60	38	
Rectum	48	27	21	
Tumor size				0.020*
<5 cm	69	48	21	
≥5 cm	77	39	38	
Tumor differentiation				0.136
Poor	34	24	10	
Well/Moderate	112	63	49	
TNM stage				0.009*
I-II	88	60	28	
III	58	27	31	
Metachronous metastasis				0.046*
Absent	107	69	38	
Present	39	18	21	

Abbreviations: TBL1XR1, Transducin (β)-like 1 X-linked receptor 1; CRC, colorectal cancer; CRCLM, colorectal cancer liver metastases.

**Table 5:** DFS of the stage I-III CRC patients (Cohort II).

Variables	Cases (n = 146)	5-year DFS (%)	DFS (months) Mean ± S.D.	Univariate P value	Multivariate P value
Gender				0.120	
Female	50	89.0%	105.3 ± 6.1		
Male	96	82.7%	91.6 ± 4.2		
Age (year)				0.647	
<60	73	87.4%	98.6 ± 4.9		
≥60	73	81.5%	94.5 ± 5.7		
Preoperative CEA level				0.078	
<100 ng/ml	70	88.2%	101.6 ± 4.2		
≥100 ng/ml	76	81.0%	90.8 ± 5.6		
Tumor location				0.063	
Colon	98	86.6%	102.3 ± 4.4		
Rectum	48	80.6%	89.7 ± 5.0		
Tumor size				<0.001*	0.001*
<5 cm	69	90.4%	102.5 ± 5.2		
≥5 cm	77	76.6%	85.0 ± 4.9		
Tumor differentiation				0.591	
Poor	34	84.6%	98.0 ± 6.4		
Well/Moderate	112	84.3%	95.9 ± 4.6		
TNM stage				0.174	
I-II	88	85.5%	99.7 ± 4.4		
III	58	83.1%	83.2 ± 5.0		
Adjunctive chemotherapy				0.869	
No	100	82.0%	96.9 ± 4.4		
Yes	46	91.2%	87.8 ± 5.0		
TBL1XR1 expression				<0.001*	<0.001*
Low	87	93.2%	106.4 ± 4.1		
High	59	70.6%	77.1 ± 5.4		

Abbreviations: TBL1XR1, Transducin (β)-like 1 X-linked receptor 1; CRC, colorectal cancer.
