# Supplementary material for: Correlations between TBL1XR1 and recurrence of colorectal cancer
Source: Sci Rep. 2017 Mar 15;7:44275. doi: 10.1038/srep44275 (PMC5353619; doi:10.1038/srep44275)
Supplement: Supplemental Tables [file srep44275-s1.doc]

**Correlations between TBL1XR1 and recurrence of colorectal cancer**

Hongda Liu^1^, *, Yunfei Xu^1^, *, Qun Zhang^2^, Kangshuai Li^1^, Dawei Wang^3^, Shuo Li^4^, Shanglei Ning^1^, Hui Yang^5^, Weichen Shi^6^, Zhaochen Liu^1^, Yuxin Chen^1, #^

1. Department of General Surgery, Qilu Hospital Affiliated to Shandong University, Jinan, Shandong 250012, China.

2. Department of Respiratory Medicine, Jinling Hospital, School of Medicine, Nanjing University, Nanjing 210002, China.

3. Department of Physiology and Pathophysiology, Peking University Health Science Center, Beijing, 100191, China

4. 302 Military Hospital of China, Beijing 100000, China.

5. Department of Gastrointestinal Surgery, Qianfoshan Hospital Affiliated to Shandong University, Jinan, Shandong 250012, China.

6. Department of Breast Surgery, Qianfoshan Hospital Affiliated to Shandong University, Jinan, Shandong 250012, China.

* Dr. Hongda Liu and Dr. Yunfei Xu contribute equally to this work.

# Correspondence to: Dr. Yuxin Chen, M.D, Ph.D

Department of General Surgery, Qilu Hospital Affiliated to Shandong University, 107 Wenhua Xi Road, Jinan, Shandong 250012, China

Email: chenyuxin_sdu@163.com

**Running title:** Prognostic role of TBL1XR1 in colorectal cancer

**Supplemental Table 1**. Disease-free survival of the stage I-II CRC patients

| Variables | Cases  (n=88) | 5-year  DFS (%) | DFS (months) Mean ± S.D. | Univariate  P value | Multivariate P value | |
| --- | --- | --- | --- | --- | --- | --- |
| Gender |  |  |  | 0.421 |  |  |
| Female | 27 | 87.1% | 104.2 ± 7.7 |  |  |  |
| Male | 61 | 85.0% | 95.8 ± 4.8 |  |  |  |
| Age (year) |  |  |  | 0.799 |  |  |
| < 60 | 46 | 88.3% | 100.8 ± 5.5 |  |  |  |
| ≥ 60 | 42 | 81.9% | 98.2 ± 7.1 |  |  |  |
| Preoperative CEA level |  |  |  | 0.140 |  |  |
| < 100 ng/ml | 45 | 87.5% | 104.8 ± 4.7 |  |  |  |
| ≥ 100 ng/ml | 43 | 83.0% | 92.7 ± 6.8 |  |  |  |
| Tumor location |  |  |  | 0.057 |  |  |
| Colon | 58 | 85.3% | 101.8 ± 4.9 |  |  |  |
| Rectum | 30 | 86.0% | 81.3 ± 6.3 |  |  |  |
| Tumor size |  |  |  | <0.001* | 0.002* |  |
| < 5 cm | 41 | 89.7% | 112.2 ± 5.1 |  |  |  |
| ≥ 5 cm | 47 | 76.3% | 86.8 ± 5.9 |  |  |  |
| Tumor differentiation |  |  |  | 0.633 |  |  |
| Poor | 25 | 87.2% | 102.2 ± 6.9 |  |  |  |
| Well/Moderate | 63 | 84.7% | 98.7 ± 5.4 |  |  |  |
| TBL1XR1 expression |  |  |  | <0.001* | <0.001* |  |
| Low | 60 | 94.3% | 111.7 ± 4.4 |  |  |  |
| High | 28 | 66.9% | 73.6 ± 6.8 |  |  |  |

Abbreviations: TBL1XR1, Transducin (β)-like 1 X-linked receptor 1; CRC, colorectal cancer.

**Supplemental Table 2**. Disease-free survival of the stage III CRC patients

| Variables | Cases  (n=58) | 5-year  DFS (%) | DFS (months)  Mean ± S.D. | Univariate  P value |
| --- | --- | --- | --- | --- |
| Gender |  |  |  | 0.074 |
| Female | 23 | 91.7% | 99.8 ± 7.0 |  |
| Male | 35 | 78.5% | 76.6 ± 5.5 |  |
| Age (year) |  |  |  | 0.788 |
| < 60 | 27 | 85.4% | 79.4 ± 7.4 |  |
| ≥ 60 | 31 | 81.2% | 83.7 ± 6.7 |  |
| Preoperative CEA level |  |  |  | 0.333 |
| < 100 ng/ml | 25 | 91.2% | 89.4 ± 7.4 |  |
| ≥ 100 ng/ml | 33 | 83.6% | 77.6 ± 6.1 |  |
| Tumor location |  |  |  | 0.699 |
| Colon | 40 | 90.3% | 79.9 ± 4.5 |  |
| Rectum | 18 | 69.7% | 82.3 ± 9.0 |  |
| Tumor size |  |  |  | 0.141 |
| < 5 cm | 28 | 87.8% | 92.7 ± 6.3 |  |
| ≥ 5 cm | 30 | 75.7% | 74.7 ± 6.0 |  |
| Tumor differentiation |  |  |  | 0.607 |
| Poor | 9 | 77.8% | 86.2 ± 14.6 |  |
| Well/Moderate | 49 | 83.8% | 78.2 ± 4.0 |  |
| Adjunctive chemotherapy |  |  |  | 0.002* |
| No | 12 | 35.7% | 48.5 ± 5.6 |  |
| Yes | 46 | 85.5% | 87.8 ± 5.0 |  |
| TBL1XR1 expression |  |  |  | 0.266 |
| Low | 27 | 90.3% | 87.6 ± 6.0 |  |
| High | 31 | 75.5% | 66.2 ± 4.0 |  |

Abbreviations: TBL1XR1, Transducin (β)-like 1 X-linked receptor 1; CRC, colorectal cancer.

**Supplemental Table 3**. Disease-free survival of the stage I-IV CRC patients without adjunctive chemotherapy

| Variables | Cases  (n=110) | 5-year  DFS (%) | DFS (months)  Mean ± S.D. | Univariate  P value |
| --- | --- | --- | --- | --- |
| Gender |  |  |  | 0.319 |
| Female | 36 | 79.9% | 97.6 ± 7.9 |  |
| Male | 74 | 74.1% | 86.5 ± 5.1 |  |
| Age (year) |  |  |  | 0.117 |
| < 60 | 53 | 85.6% | 98.6 ± 5.5 |  |
| ≥ 60 | 57 | 65.9% | 83.6 ± 7.1 |  |
| Preoperative CEA level |  |  |  | 0.075 |
| < 100 ng/ml | 54 | 80.7% | 97.9 ± 5.4 |  |
| ≥ 100 ng/ml | 56 | 71.0% | 83.2 ± 6.6 |  |
| Tumor location |  |  |  | 0.083 |
| Colon | 70 | 80.7% | 96.7 ± 5.3 |  |
| Rectum | 40 | 69.4% | 82.5 ± 6.2 |  |
| Tumor size |  |  |  | 0.001 |
| < 5 cm | 50 | 84.6% | 103.7 ± 6.2 |  |
| ≥ 5 cm | 60 | 69.6% | 78.6 ± 5.7 |  |
| Tumor differentiation |  |  |  | 0.861 |
| Poor | 30 | 75.8% | 91.7 ± 7.8 |  |
| Well/Moderate | 80 | 75.8% | 90.9 ± 5.5 |  |
| TNM stage |  |  |  | <0.001* |
| I-II | 88 | 88.6% | 99.7 ± 4.4 |  |
| III-IV | 22 | 19.7% | 36.0 ± 4.9 |  |
| TBL1XR1 expression |  |  |  | <0.001* |
| Low | 63 | 91.3% | 98.4 ± 4.8 |  |
| High | 47 | 60.5% | 63.7 ± 6.0 |  |

Abbreviations: TBL1XR1, Transducin (β)-like 1 X-linked receptor 1; CRC, colorectal cancer.
